# Simultaneous Determination of Human Serum Albumin and Low-Molecular-Weight Thiols after Derivatization with Monobromobimane

**DOI:** 10.3390/molecules26113321

**Published:** 2021-06-01

**Authors:** Katarzyna Kurpet, Rafał Głowacki, Grażyna Chwatko

**Affiliations:** 1 Doctoral School of Exact and Natural Sciences, University of Lodz, Banacha 12/16, 90-237 Lodz, Poland; 2 Department of Environmental Chemistry, Faculty of Chemistry, University of Lodz, Pomorska 163, 90-236 Lodz, Poland; rafal.glowacki@chemia.uni.lodz.pl

**Keywords:** low-molecular-weight thiols, human serum albumin, α-lipoic acid, blood plasma, derivatization, monobromobimane, reduction, sodium borohydride, high-performance liquid chromatography, fluorescence detection

## Abstract

Biothiols are extremely powerful antioxidants that protect cells against the effects of oxidative stress. They are also considered relevant disease biomarkers, specifically risk factors for cardiovascular disease. In this paper, a new procedure for the simultaneous determination of human serum albumin and low-molecular-weight thiols in plasma is described. The method is based on the pre-column derivatization of analytes with a thiol-specific fluorescence labeling reagent, monobromobimane, followed by separation and quantification through reversed-phase high-performance liquid chromatography with fluorescence detection (excitation, 378 nm; emission, 492 nm). Prior to the derivatization step, the oxidized thiols are converted to their reduced forms by reductive cleavage with sodium borohydride. Linearity in the detector response for total thiols was observed in the following ranges: 1.76–30.0 mg mL^−1^ for human serum albumin, 0.29–5.0 nmol mL^−1^ for α-lipoic acid, 1.16–35 nmol mL^−1^ for glutathione, 9.83–450.0 nmol mL^−1^ for cysteine, 0.55–40.0 nmol mL^−1^ for homocysteine, 0.34–50.0 nmol mL^−1^ for N-acetyl-L-cysteine, and 1.45–45.0 nmol mL^−1^ for cysteinylglycine. Recovery values of 85.16–119.48% were recorded for all the analytes. The developed method is sensitive, repeatable, and linear within the expected ranges of total thiols. The devised procedure can be applied to plasma samples to monitor biochemical processes in various pathophysiological states.

## 1. Introduction

Thiols constitute a class of organic sulfur compounds characterized by the presence of a sulfhydryl functional group (–SH), also known as a thiol group [1,2,3,4]. In a biological system, thiols are present as albumin thiols, protein-bound thiols, and low-molecular-weight thiols such as cysteine (Cys), homocysteine (Hcy), glutathione (GSH), and cysteinylglycine (Cys-Gly) [2,4,5,6]. These free thiols are metabolically related [4,5,6]. Hcy can be catalyzed to Cys, which in turn is a precursor of GSH—a highly important tripeptide in biological terms. Cys-Gly is an intermediate metabolite in GSH metabolism and is the second most abundant low-molecular-weight thiol in human plasma after Cys [5,7,8]. Reduced, free oxidized, and protein-bound thiols, i.e., Cys, Hcy, Cys-Gly, and GSH, comprise an antioxidant buffer that maintains the reduction–oxidation balance inside the cell and tissues [9,10]. Under physiological conditions, thiols are strong reductants that can undergo reversible or irreversible oxidation processes through one- or two-electron reaction mechanisms, resulting in a wide range of products [11]. Some of them, including disulfides, can be reverted to free thiols in the presence of suitable reducing agents. Such thiol–disulfide homeostasis plays a role in cellular defense against toxic substances, free radicals, and reactive oxygen species, as well as in apoptosis, transcription, enzyme activity regulation, and in the maintenance of the proper structure and function of proteins [5,12,13].

Other biological aminothiols that widely occur in animal tissues and fluids include N-acetyl-L-cysteine (NAC) and α-lipoic acid (α-LA). The former, NAC, is exclusively present in urine and derives from N-acetylation of Cys in the kidney [14,15]. This compound is a commonly used mucolytic drug that alleviates mucus retention by reducing highly cross-linked mucus glycoproteins [3,4,16,17,18,19]. In addition, NAC increases the activity of glutathione S-transferase, has anti-inflammatory effects, can break down the pathogenic biofilm, and is widely used in the treatment of acetaminophen overdose. Moreover, the administration of NAC provides Cys as a substrate for the intracellular synthesis of GSH, which is one of the most important naturally occurring antioxidants. The drug may circulate in a free or protein-bound form in plasma, exhibiting the half-life of several dozen minutes after oral administration, which is due to extensive first-pass metabolism in the body [18,20]. Interestingly, NAC causes a substantial decline in plasma low-molecular-weight thiols, including Cys, Cys-Gly, and Hcy, by increasing urinary excretion [21]. NAC administration is also beneficial in systemic sclerosis, cancer chemotherapy, HIV infection, and septic shock [22]. 

The second aminothiol mentioned above, α-LA, is a naturally occurring cofactor of several multienzyme complexes involved in energy production [23,24,25]. It can be synthesized from octanoic acid and Cys, but the quantity produced is negligible. In human cells, α-LA is reduced to dithiol, i.e., dihydrolipoic acid, which has two thiol groups per molecule derived from a dithiolane ring. Owing to such a structure, this compound maintains its protective functions in both oxidized and reduced forms [22,24]. *De novo* synthesis is not the only source of α-LA in mammalian cells. The body acquires it with food, mainly of animal origin. This acid exists naturally– via a covalent amide bond—in conjunction with the ε-amino group of lysine. Together, they form lipoyllysine (LLYS), which—when taken with food—can be hydrolyzed in the blood to α-LA by the enzyme called lipoamidase [26]. Even though the content of LLYS in food is sufficient for metabolic processes to take place, the concentration of α-LA that can be obtained during the hydrolysis of this compound in the blood is insufficient for therapeutic purposes [27]. Therefore, α-LA is an extensively used nutraceutical to combat the negative effects of oxidative stress since it meets all the criteria for a perfect antioxidant. It is applicable in various fields of medicine, including in the treatment of diabetic neuropathy, neurodegenerative and cardiovascular diseases, as well as in the fight against obesity, poisoning, cancer, or body aging [25,27]. The half-life of α-LA elimination from the blood, regardless of dose and method of administration, is approximately 30 min [28].

The human plasma has relatively low concentrations of low-molecular-weight thiols but is characterized by the presence of human serum albumin (HSA) as the most abundant protein thiol [11]. The main biological roles of HSA include regulation of plasma pressure and transport of various ligands, such as drugs, hormones, xenobiotics, fatty acids, and metal ions [29,30,31]. Moreover, HSA is an effective extracellular antioxidant. It scavenges reactive oxygen species, which are responsible for the development of many diseases associated with oxidative stress [30]. The antioxidant activity of HSA results from the presence of a thiol group derived from Cys-34, which is not involved in the formation of intra-chain disulfide bonds and comprises approximately 80% of total free thiols in plasma [1,29,30,31]. HSA has been used for over 50 years to treat several conditions related to hypovolemia [11]. In addition, this protein is also of interest to pharmacists as a drug carrier.

Altered thiol levels in physiological fluids have been associated with specific pathological conditions and are closely related to several human diseases, especially premature atherosclerosis [32], vascular disease [33], diabetes [34], cancer [35], rheumatoid arthritis [36], leukemia [14], chronic kidney disease [37], acquired immunodeficiency syndrome [32], multiple sclerosis [12], amyotrophic lateral sclerosis [12], and neurodegenerative diseases [12,38] such as Parkinson’s and Alzheimer’s disease. Due to the importance of the dynamic thiol–disulfide homeostasis and the potential use of plasma levels as valuable information about specific pathological conditions associated with several human diseases, there is a need for measurements of aminothiols in biological samples to understand their physiological role. Nevertheless, the development of highly sensitive and selective analytical methods is difficult since biological thiols do not have specific physicochemical properties which are required for high detection sensitivity. Moreover, thiols are unstable in isolated plasma and have a tendency to oxidize to disulfides. Another challenge in the analysis of thiols is that the high polarity and water solubility of these compounds make them difficult to extract from complex biological matrices such as human blood plasma.

Despite these difficulties, numerous methods have been described for the determination of total thiols in biological fluids. The most commonly used techniques are high-performance liquid chromatography (HPLC) with different detection modes, mainly ultraviolet [4,5,6,30,39,40] and fluorescence [41,42,43,44,45,46,47], capillary electrophoresis [48,49], or spectrophotometry [12,50]. Although several techniques have been used to determine thiols, there are still some problems caused by the need for complicated instrumentation, complexity of the procedure, the time-consuming nature of the methods, and the number of thiols quantified simultaneously [14,30,51,52]. Among the available techniques, the most commonly used method for total thiol determination—due to its high selectivity and sensitivity—is the approach based on pre- or post-column derivatization followed by separation and fluorescence detection. For this reason, various types of labeling reagents such as bimanes, ortho-phthalaldehyde, N-substituted maleimides, halides, and halogen-benzofurazans have been developed for the selective determination of sulfhydryl group-containing compounds. Since concentrations of total thiols are important biomarkers, the development of new fluorescence labeling reagents for more sensitive thiol detection is ongoing.

**Figure 1:**
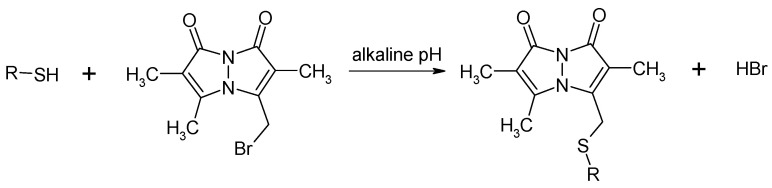
General equation of a chemical derivatization reaction of thiols with mBBr.

A new reversed-phase (RP) HPLC-based method for the simultaneous determination of HSA, α-LA, NAC, and metabolically related major plasma low-molecular-weight aminothiols Cys, GSH, Hcy, and Cys-Gly is described herein. The method is based on the derivatization (Figure 1) of analytes with a thiol-specific labeling reagent, monobromobimane (mBBr), and fluorescence detection of stable derivatives at the excitation and emission wavelengths of 378 nm and 492 nm, respectively. The disulfides are converted to their reduced counterparts by reductive cleavage with sodium borohydride (NaBH_4_) prior to the derivatization step. The usefulness of the developed method has been proven by its application in real plasma samples from 10 apparently healthy individuals.

The mBBr has been previously used for the determination of low-molecular-weight aminothiols [41,43,53,54,55,56]. Now, its application has been extended to the derivatization of proteins such as albumin. The novelty of the presented procedures consists in the development of new chromatographic conditions for simultaneous determination of low-molecular-weight aminothiols and albumin and demonstration of the short-term stability of thiol-bimane adducts at acidic pH.

## 2. Results and Discussion

### 2.1. Sample Preparation

Determination of thiols in biological samples is an extremely difficult task due to their high oxidoreductive activity. Moreover, most endogenous thiols lack the structural properties necessary to provide a signal compatible with standard detectors such as spectrophotometry or fluorescence. If these detection methods are used, an additional sample preparation step, i.e., derivatization, must be initiated. However, this involves a longer overall analytical process and is associated with the risk of increasing the overall analytical error. In biological systems, thiols occur mainly in oxidized or protein-bound forms, and therefore the reduction of disulfide bonds to thiol groups must be performed prior to the derivatization reaction. NaBH_4_ [5,41,54], dithiothreitol (DTT) [42,43,52], tris(2-carboxyethyl)phosphine (TCEP) [4,30,39,57], or 2-mercaptoethanol (2-ME) [6,52] are the most commonly used substances to reduce disulfide bonds. The choice of an appropriate reductant depends on the analytical procedure to be followed, especially on the type of the derivatizing reagent used and the detection method. The use of reagents containing a sulfhydryl group, such as 2-ME or DTT, is almost impossible when using a derivatizing reagent that is highly selective towards the thiol group. The derivatives formed lead to increased consumption of the derivatizing reagent, which can lead to underestimation of the results. In addition, these derivatives interfere with the peaks of the analytes by forming additional very large signals in the chromatogram, making interpretation difficult. Limitations in the use of trialkyl phosphines relate primarily to their toxicity, but also to their irritating and pungent odor. Moreover, the most commonly used phosphine, TCEP, can interfere with certain derivatizing agents such as mBBr [58,59] or 5,5′-dithiobis-2-nitrobenzoic acid [60]. NaBH_4_ seems to be the optimal reducing reagent. It is non-toxic, readily available, and the excessive amount can be easily and quickly removed by adding hydrochloric acid or acetone. The major advantage of this reducing agent is the short reaction time at high reagent concentrations. Since NaBH_4_ is unstable in aqueous solutions, it must be prepared immediately before use. The addition of an organic solvent such as dimethyl sulfoxide (DMSO) increases the stability of NaBH_4_. The main disadvantage of using NaBH_4_ is its tendency to form aerosols during the reaction due to the intense release of hydrogen. However, this phenomenon can be eliminated by the addition of surfactants such as n-octanol. The thiols obtained in the reduction step can be re-oxidized, so the resulting product should be immediately subjected to a derivatization reaction to block the labile sulfhydryl group. Additionally, the metal cation-catalyzed oxidation of thiols to disulfides can be slowed down by adding a complexing compound such as ethylenediaminetetraacetic acid disodium salt (EDTA-Na_2_) to the sample. In HPLC coupled with fluorescence detection, such derivatizing reagents as mBBr [41,43,53,54,55,56], ammonium 4-fluoro-2,1,3-benzoxadiazole-7-sulfonate [46,47], 4-aminosulfonyl-7-fluoro-2,1,3-benzoxadiazole [44,45], o-phthalaldehyde [61,62], or 4-bromomethyl-6,7-dimethoxycoumarin [63,64], have been widely used for the derivatization of thiols in biological samples. Most of these reagents have an active halogen in their structure, which is exchanged for the sulfur atom of the thiol group of the analyte in a nucleophilic substitution reaction to form a stable thioether.

#### 2.1.1. Disulfide Bonds Cleavage

The determination of total thiol requires the reduction of disulfide bonds since most low-molecular-weight plasma thiols are oxidized in the form of symmetrical or mixed disulfides and bound to proteins, mainly albumin. This fact makes the thiol groups inaccessible to the derivatizing reagent, and therefore plasma samples must be treated with a reducing reagent to release the free thiols. NaBH_4_ was used for this purpose. We examined the time and molar excess of the borohydride response curves to determine the conditions necessary for complete reduction of the disulfide bonds. Maximum reduction was achieved after five minutes of incubation at room temperature. The highest peak areas for all the analytes were observed when 30 µL of 6 mol L^−1^ NaBH_4_ were used for the reduction (data not shown). The plasma Hcy, Cys-Gly, and Cys assay methods developed by Fiskerstrand et al. [53] used a lower concentration of NaBH_4_ (4 mol L^−1^) for a 3-min reduction, but the addition of dithioerythritol was necessary for maximum yield of all thiol components. Another approach was used by Mansoor et al. [41], where samples were incubated simultaneously with 2 mol L^−1^ NaBH_4_ and mBBr for 10 min at room temperature in the dark while total plasma thiols were determined. In both methods, the volume of NaBH_4_ added to the sample was also 30 µL. In our method, 6 µL of 3 mol L^−1^ HCl were added to the thiol-containing sample to remove excess reductant. To avoid foaming of the reaction mixture due to the strong hydrogen evolution, 20 µL of n-octanol were added to the plasma samples according to the previously described methods [41,53]. These reduction conditions were adopted for a routine plasma analysis for total thiols, including HSA.

#### 2.1.2. Derivatization via the –SH Group

**Figure 2:**
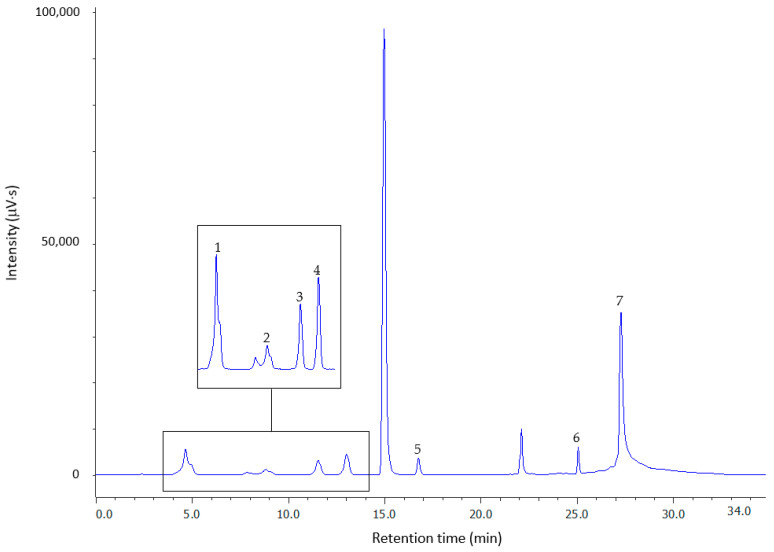
Representative chromatogram for total forms of 10 nmol mL^−1^: Cys (1), Cys-Gly (2), Hcy (3), GSH (4), NAC (5), α-LA (6), and 3 mg mL^−1^ HSA (7) in standard water solutions after reduction with NaBH_4_ and derivatization with mBBr. Chromatographic conditions as described in Section 3.2. The unsigned peaks are derived from mBBr and its hydrolysis reaction products.

Since thiols do not contain a fluorophore in their structure that allows their direct fluorimetric detection, it is necessary to perform a derivatization reaction during the sample preparation step. For this purpose, we used mBBr, which reacts violently with thiols at alkaline pH and room temperature. The resulting highly fluorescent and stable thioethers can be easily detected, even at low analyte concentrations. The reagent itself also fluoresces and its peak is visible in the chromatogram along with the mBBr hydrolysis reaction products (Figure 2). Monobromobimane is readily photodegraded, so it is necessary to protect it from light and perform the reaction in the dark. The yield of the derivatization reaction was optimized with respect to pH (Figure S1), excess reagent (Figure S2), and time (Figure S3). The results indicate that the optimum reaction pH for the derivatization of endogenous plasma thiols with mBBr occurred at pH 9.5, and the reaction was completed after 10 min with 70-fold excess reagent in the dark and at room temperature. The obtained conditions are similar to those described earlier, where the reaction was carried out in *N*-ethylmorpholine at pH 9 for 10 min [41] or 20 min [54].

**Figure 3:**
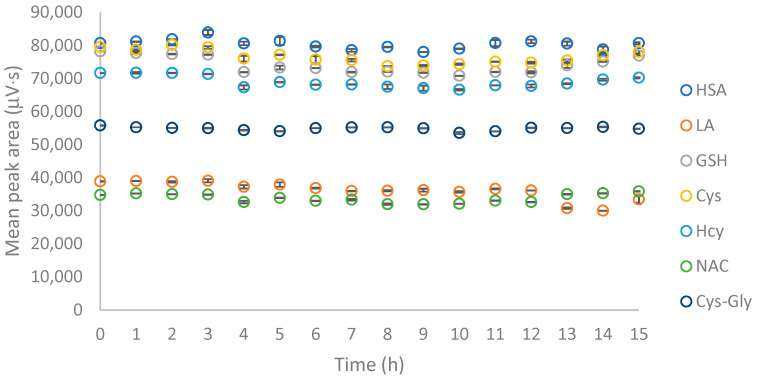
Stability of the analyzed thiol–bimane derivatives in an acidic environment, *n* = 3. In the figure, the standard deviation bars of peak areas are presented.

Finally, the derivatization reaction was quenched by adding 200 μL of 1 mol L^−1^ HCl to reach pH of 3. Other acids used to complete the derivatization of low-molecular-weight thiols include perchloric acid [41] or glacial acetic acid [53], however, their use in this procedure would precipitate HSA. The short-term stability of thiol–bimane adducts is not described in the literature, therefore it was tested during the research. For all the analytes, the thiol–bimane derivatives were stable in an acidic environment at room temperature over the time period studied (Figure 3). This means that the final solution kept at room temperature in the autosampler can be safely analyzed for up to 15 h which allows for long unattended runs.

### 2.2. Chromatography

Preliminary experiments aimed at developing a method for the simultaneous determination of low-molecular-weight thiols and HSA in plasma were performed in standard water solutions. In order to achieve good separation of the analyzed thiol–bimane adducts, various parameters of the chromatographic conditions were tested, including the concentration of trifluoroacetic acid (TFA), acetonitrile (ACN) content, pH of the mobile phase, concentration of the organic modifier, and gradient profile. The experiments mentioned briefly above led to the establishment of optimum chromatographic conditions for all the analytes, which are described in Section 3.2. Under these conditions, the thiol–bimane derivatives eluted after 5.14 min (RSD, 0.86%, *n* = 3) for Cys, 9.63 min (RSD, 0.77%, *n* = 3) for Cys-Gly, 12.30 min (RSD, 0.61%, *n* = 3) for Hcy, 13.76 min (RSD, 0.42%, *n* = 3) for GSH, 17.07 min (RSD, 0.74%, *n* = 3) for NAC, 25.08 min (RSD, 0.07%, *n* = 3) for α-LA, and 27.82 min (RSD, 0.05%, *n* = 3) for HSA. As can be seen from the chromatogram in Figure 2, the four bimane derivatives of low-molecular-weight thiols elute in pairs: GSH in close proximity to Hcy and Cys in close proximity to Cys-Gly, but in this case, the peaks are separated by a reagent peak. These results are in agreement with those obtained for CMQT derivatives [5,30]. The longer hydrophobic chain of NAC and reduced α-LA causes these compounds to elute later than the remaining small thiols. The HSA derivative exhibits the highest hydrophobicity and elutes last. The retention factors for all the analytes varied depending on the concentration and pH of the TFA used in the aqueous phase as a consequence of a change in the hydrophobicity of the eluent and the degree of ionization of the analyte. Despite the high particle size discrepancy of the analyzed biothiols, optimization of the separation conditions resulted in the good separation of the mBBr derivatives of Cys, Cys-Gly, Hcy, GSH, NAC, α-LA, and HSA.

### 2.3. Method Validation

The method was validated according to the guidelines for the analysis of biological samples [65,66]. The validation parameters of linearity, precision, accuracy, and limits of detection (LOD) and quantification (LOQ) were checked and tested. The standard addition method was used to calibrate the method.

#### 2.3.1. Linearity

The linearity of the method was determined by analyzing plasma samples spiked with the standard solution of thiols prepared as described in Section 3.5.1. Six-point calibration curves were prepared for six low-molecular-weight thiols and HSA in triplicate (Appendix A). Each sample was processed according to the recommended procedure. The validation parameters for all fitted calibration plots were satisfactory. Detailed method calibration data are presented in Table 1.

#### 2.3.2. Limits of Detection and Quantification

LOD is defined as the lowest concentration of the analyte that can be detected with a given measuring device, but without its quantitative determination. LOQ is the lowest concentration of a substance that can be quantified with a given analytical method with the assumed accuracy and precision. The measured validation parameters were evaluated by applying the calibration standard of thiols in proxy matrix (0.9% NaCl in 10 mmol L^−1^ phosphate buffer, pH 7.4) solutions obtained on this occasion. LOD was calculated according to the following formula:

(1) $$LOD=\frac{3.3\;s}{b}$$

where *b* is the slope of the calibration curve, and *s* is the standard deviation of the intercept of the regression line.

LOQ values for the analytes tested were calculated based on the LOD using the equation below:

(2) LOQ=3LOD

The limits of detection and quantification for HSA, Cys, Hcy, Cys-Gly, NAC, α-LA, and GSH are presented in Table 1.

#### 2.3.3. Precision and Accuracy

To assess the quality of the devised method for the simultaneous determination of plasma thiols, including HSA, precision and accuracy were determined. The intra- and inter-day precision and accuracy values were measured in triplicate in plasma samples spiked with standard solutions of thiols to obtain four concentrations representing the full range of calibration curves. The measured concentrations were determined by applying the calibration curves obtained on this occasion. Precision was expressed as relative standard deviation (RSD) while accuracy was expressed as percentage recovery of the analyte using the following formula:

(3) $$Accuracy\;\left(\%\right)=\frac{measured\;amount-endogenous\;content}{added\;amount}\times 100\%$$

The inter-day precision and recovery values were evaluated on three consecutive days in a week. Detailed intra-day and inter-day precision and accuracy data are presented in Table 2. The estimated validation parameters met the requirements applicable to the analysis of biological samples.

#### 2.3.4. Matrix Effect

The matrix effect was evaluated using the slope comparison method. First, the slope coefficients of the calibration curves obtained for thiol standard solutions and the calibration curves obtained for a plasma sample spiked with a known amount of the analyte were compared. The differences between the slope coefficients of the calibration curves for each analyte are presented with reference to RSD, which is, respectively, 34.50% for HSA, 16.86% for α-LA, 11.18% for Cys, 19.73% for Hcy, 15.71% for GSH, 16.24% for NAC, and 141.30% for Cys-Gly. These data indicate that there was a significant matrix effect on the results of endogenous thiol determination in plasma samples. Next, we investigated whether the matrix effect on assay results occurred between plasma samples from five different subjects. The RSD for the coefficients of the calibration curves was as follows: 54.31% for HSA, 31.75% for α-LA, 15.87% for GSH, 17.16% for Cys, 20.50% for Hcy, 26.55% for NAC, and 23.10% for Cys-Gly. Furthermore, in this case, the obtained results prove the existence of the matrix effect in the determination of thiol compounds in plasma samples collected from different individuals. Such an excessive impact of the matrix may result from the fact that plasma samples from randomly selected individuals of different ages were used in the study, without preselection of the subjects for medications, chronic diseases, and other factors that may influence the content of the tested compounds in the bloodstream.

### 2.4. Application to Real Plasma Samples

**Figure 4:**
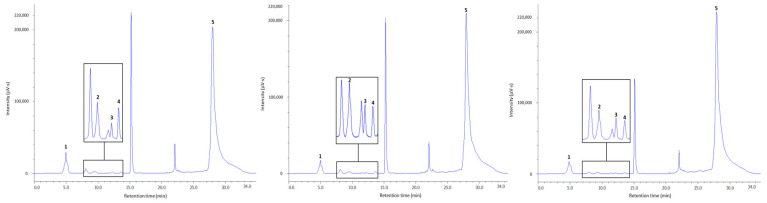
Chromatograms of plasma samples derived from potentially healthy volunteers (from the left: Nos. 1, 3, and 9 Figure 3. after reduction with NaBH_4_ and derivatization with mBBr. The peaks labeled 1, 2, 3, 4, and 5 correspond to the thiol–bimane signals Cys, Cys-Gly, Hcy, GSH, and HSA, respectively. Chromatographic conditions as described in Section 3.2. The unsigned peaks are derived from mBBr and its hydrolysis reaction products.

The devised and validated method was applied to the simultaneous determination of HSA and low-molecular-weight thiols in plasma samples from 10 apparently healthy subjects (Figure 4). Due to the matrix effect, the method of simple standard addition was used for the determination of analytes [67]. The concentration of the analyte in each sample was calculated using the following formula:

(4) $$C_{x}=\frac{Y_{x}\times C_{s}}{Y_{s}-Y_{x}}$$

where *C_x_*—concentration of the analyte in the plasma sample; *Y_x_*—analytical signal for the sample containing only the analyte; *C_s_*—concentration for the sample with the addition of a known amount of the standard; *Y_s_*—analytical signal for the sample with the addition of a known amount of the standard.

The expected concentration ranges for total thiols in human plasma were as follows: 3.27–13.37 nmol mL^−1^ for Hcy [56], 135.80–266.50 nmol mL^−1^ for Cys [56], 19.24–39.74 nmol mL^−1^ for Cys-Gly [56], 4.90–7.30 nmol mL^−1^ for GSH [11], 35.9–48.3 mg mL^−1^ for HSA [30], and 1.33–5.8 nmol mL^−1^ for α-LA (in the case of administration of 20 or 100 mg LA supplement) [30,45]. We also expected that, without administration, NAC could not be detected in plasma from healthy volunteers at a concentration exceeding the method’s detection limit as reported earlier [15]. The mean total thiol content ± standard deviation (SD) in human plasma was as follows: 23.98 ± 1.60 mg mL^−1^ for HSA, 62.99 ± 4.31 nmol mL^−1^ for Cys, 3.93 ± 0.28 nmol mL^−1^ for Hcy, 5.29 ± 0.31 nmol mL^−1^ for GSH, and 50.20 ± 3.28 nmol mL^−1^ for Cys-Gly. The concentrations of α-LA and NAC were not detected in the plasma samples examined. This may be due to the fact that the volunteers did not take specimens containing these compounds. Detailed analytical data for total Cys, Hcy, GSH, Cys-Gly, and HSA are shown in Table 3. These results are mostly in agreement with those previously reported [5,11,30,39,56,57]. The elaborated method can be successfully applied in large populations to monitor changes in thiol concentration in different physiological conditions.

## 3. Materials and Methods

### 3.1. Chemicals and Reagents

Dimethyl sulfoxide (DMSO), 1-octanol, trifluoroacetic acid (TFA), oxidized glutathione (GSSG), cysteinylglycine (Cys-Gly), homocysteine (Hcy-Hcy), *N*-acetyl-l-cysteine (NAC), α-lipoic acid (LA), human serum albumin (HSA), and monobromobimane (mBBr) were obtained from Sigma Aldrich (St. Louis, MO, USA). l-cystine (Cys-Cys) was purchased from Reanal (Budapest, Hungary). Ethylenediaminetetraacetic acid disodium salt (EDTA-Na_2_), sodium hydroxide (NaOH), and HPLC-grade acetonitrile (ACN) were bought from J.T. Beaker (Deventer, The Netherlands). Hydrochloric acid (HCl) was supplied by POCH (Gliwice, Poland). Sodium borohydride (NaBH_4_) was delivered by Merck (Darmstadt, Germany), and 2-amino-2-(hydroxymethyl)-1,3-propanediol (Tris base) was from BioShop (Canada). Deionized water was prepared in our laboratory using the Mili-QRG system (Millipore, Vienna, Austria). The pH of the buffers was adjusted by potentiometric titration using a system calibrated with standard pH solutions.

### 3.2. HPLC Instrumentation and Chromatographic Conditions

All the analyses were performed on an integrated LC-4000 Series JASCO RHPLC system (JASCO, Tokyo, Japan) equipped with a quaternary pump (model No. PU-4180, Tokyo, Japan), a vacuum degasser, an autosampler (model No. AS-4150, Tokyo, Japan), a column oven (model No. CO-4062, Tokyo, Japan), and a fluorescence detector (model No. FP-4020, Tokyo, Japan) operating at the excitation and emission wavelengths of 378 nm and 492 nm, respectively. The detector signal was amplified tenfold. System control and data acquisition processes were performed using the ChromNAV2 software. Spectra Manager ver. 2 was used to analyze the spectra.

The samples (5 μL) were injected onto a 150 × 4.6 mm, 3.6 μm particle size Aeris™ WIDEPORE XB-C18 column (Phenomenex, CA, USA) using an autosampler. The mobile phase consisted of 0.1% TFA in water (A) and 0.1% TFA in ACN (B). All the analyses were performed at room temperature and the flow rate of the mobile phase was 1 mL min^−1^. Chromatographic separation of HSA and low-molecular-weight thiols was achieved in 35 min with gradient elution as follows: 0–5 min, 5% B; 5–11 min, 5–8% B; 11–18 min, 8–15% B; 18–20 min, 15–30% B; 20–25 min, 30–40% B; 25–26 min, 40–50% B; 26–30 min, 50–70% B; 30–33 min, 70–5% B; 33–35 min, 5% B. Peaks were identified by comparing retention times and fluorescence spectra with corresponding data from the authentic standard.

### 3.3. Preparation of Stock and Buffer Solutions

Stock solutions of 0.01 mol L^−1^ GSSG, Hcy-Hcy, Cys-Gly and 0.1 mol L^−1^ Cys-Cys and NAC required for method development were prepared by dissolving appropriate amounts of the compounds in 100 μL of 1 mol L^−1^ HCl and diluting to 1 mL. A stock solution of 0.01 mol L^−1^ α-LA was prepared in 0.1 mol L^−1^ NaOH. The stock solution of HSA was prepared by dissolving 300 mg of the protein in 1 mL deionized water. These solutions were kept at 4 °C for one week without any noticeable change in the analyte content. The working solutions were prepared daily by appropriate dilutions with deionized water as needed and processed without delay.

The stock solution of the reducing agent was prepared daily by dissolving appropriate amounts of NaBH_4_ in 500 μL of 0.1 mol L^−1^ NaOH to give a concentration of 6 mol L^−1^ and then preparing a mixture of NaBH_4_ and DMSO in a volume ratio of 2:1.

The stock solution of 0.1 mol L^−1^ mBBr was prepared in ACN and stored at 4 °C in the dark for up to one month, without any noticeable change in the content.

The buffer solution of 0.2 mol L^−1^ Tris–HCl, pH 9.5, containing 0.2 mmol L^−1^ EDTA-Na_2_ was prepared by dissolving 2.4228 g Tris base in 100 mL water and adjusted with 3 mol L^−1^ HCl to pH 9.5 by potentiometric titration. Then, the stock solution of 0.02 mol L^−1^ EDTA was prepared by dissolving 0.0074448 g of the compound in a 0.2 mol L^−1^ Tris–HCl buffer solution, pH 9.5. Finally, 900 µL EDTA stock solution was mixed with 89.1 mL buffer solution for a final volume of 90 mL.

### 3.4. Human Plasma Sample Collection and Storage

Blood (2 mL) was collected by venipuncture from 10 apparently healthy subjects of various ages into vacutainer tubes containing EDTA. The tubes were immediately placed on ice and centrifuged at 3500 rpm at 4 °C for 10 min. After centrifugation, the clear plasma supernatant was collected and stored at −80 °C until analysis.

### 3.5. Analytical Method Validation

The methods were validated according to the International Conference on Harmonization (ICH) guidelines for validation of analytical procedures [66] and the Food and Drug Administration (FDA) guidelines for analytical procedures and methods validation for drugs and biologics [65].

#### 3.5.1. Preparation of Calibration Standards

To prepare calibration standards for the determination of total thiols in human plasma, 70 μL plasma from each of the apparently healthy individuals was placed in a test tube and spiked with 10 μL disulfide mixture to obtain the following concentrations: 1.76, 3.0, 5.0, 20.0, 25.0, 30.0 mg mL^−1^ plasma for HAS; 0.29, 0.5, 1.0, 2.0, 4.0, 5.0 nmol mL^−1^ plasma for α-LA; 1.16, 3.0, 5.0, 10.0, 20.0, 35.0 nmol mL^−1^ plasma for GSH; 9.83, 20.0, 35.0, 50.0, 250.0, 450.0 nmol mL^−1^ plasma for Cys; 0.55, 1.0, 3.0, 5.0, 20.0, 40.0 nmol mL^−1^ for Hcy; 0.34, 1.0, 5.0, 10.0, 30.0, 50.0 nmol mL^−1^ for NAC; and 1.45, 5.0, 10.0, 20.0, 30.0, 45.0 nmol mL^−1^ plasma for Cys-Gly. In the next step, 20 μL 1-octanol was added. Subsequently, the disulfide bonds were reduced for five minutes at room temperature by treatment with 30 μL of a mixture containing 6 mol L^−1^ NaBH_4_ in DMSO (2:1; *v*:*v*) followed by adding 6 µL of 3 mol L^−1^ HCl. After the reduction reaction ended, 657 µL of 0.2 mol L^−1^ Tris–HCl (pH 9.5) with 0.2 mmol L^−1^ EDTA buffer were added. Eventually, the thiols were derivatized with 7 µL of 0.1 mol L^−1^ mBBr for 10 min at room temperature in the dark. To stop the derivatization reaction, 200 µL of 1 mol L^−1^ HCl were added. Of the final sample, 5 µL were injected into the chromatographic column. In all cases, the calibration standards were prepared in triplicate.

#### 3.5.2. Calibration Curves

The calibration range was 1.76–30.0 mg mL^−1^ plasma for HSA, 0.29–5.0 nmol mL^−1^ plasma for α-LA, 1.16–35.0 nmol mL^−1^ plasma for GSH, 9.83–450.0 nmol mL^−1^ plasma for Cys, 0.55–40.0 nmol mL^−1^ for Hcy, 0.34–50.0 nmol mL^−1^ for NAC, and 1.45–45.0 nmol mL^−1^ plasma for Cys-Gly. The calibration curves were constructed using a linear least-squares regression model by plotting the peak area of the respective thiol mBBr derivative against the analyte concentration.

#### 3.5.3. Limits of Detection and Quantification

LOD and LOQ were determined based on the standard deviation of the intercept and the slope of the calibration curve obtained for standard solutions of the analytes. The calibration standards and curves were prepared as follows: 70 μL proxy matrix (0.9% NaCl in 10 mmol L^−1^ phosphate buffer, pH 7.4) was placed in each test tube and spiked with 10 μL disulfide mixture to obtain the following concentrations: 1.0, 3.0, 5.0, 20.0, 25.0, and 30.0 mg mL^−1^ for HSA; 0.1, 0.3, 0.5, 2.0, 4.0, and 5.0 nmol mL^−1^ for α-LA; 0.3, 1.0, 5.0, 10.0, 20.0, and 35.0 nmol mL^−1^ for GSH; 5.0, 7.0, 10.0, 50.0, 250.0, and 450.0 nmol mL^−1^ for Cys; 0.3, 0.5, 1.0, 5.0, 20.0, and 40.0 nmol mL^−1^ for Hcy; 1.0, 3.0, 5.0, 7.0, 10.0, and 50.0 nmol mL^−1^ for NAC; 0.7, 5.0, 10.0, 20.0, 30.0, and 45.0 nmol mL^−1^ for Cys-Gly. The further procedure was the same as in Section 3.5.1.

#### 3.5.4. Precision and Accuracy

Assay precision was determined intra-day and inter-day. Intra-day precision was assessed by assaying samples with the same concentration in triplicate and on the same day. Plasma samples were enriched with thiol standards at the following concentrations: 1.76, 5.0, 15.0, and 30.0 mg mL^−1^ for has; 0.29, 0.9, 1.75, and 4.0 nmol mL^−1^ for α-LA; 1.16, 4.0, 15.0, and 30.0 nmol mL^−1^ for GSH; 9.83, 30.0, 100.0, and 350.0 nmol mL^−1^ for Cys; 0.55, 1.7, 15.0, and 30 nmol mL^−1^ for Hcy; 0.34, 1.0, 20.0, and 40.0 nmol mL^−1^ for NAC; and 1.45, 4.5, 20.0, and 35.0 nmol mL^−1^ for Cys-Gly. The inter-day precision was investigated by comparing the assays on three different days. Three sample solutions with the same concentration as above were prepared and assayed in triplicate.

#### 3.5.5. Matrix Effect Evaluation

The slope comparison method was used to evaluate the matrix effect. First, the slopes of the standard curves in plasma were compared with the slopes of the standard curves in a proxy matrix (0.9% NaCl in 10 mmol L^−1^ phosphate buffer, pH 7.4). In this case, the matrix effect was determined by spiking 70 μL of plasma or proxy matrix with 10 μL of mixed standard solutions to obtain low, medium, and high analyte concentrations as follows: 7.0, 50.0, and 250.0 nmol mL^−1^ for Cys; 2.0, 10.0, and 30.0 nmol mL^−1^ for Cys-Gly; 0.5, 5.0, and 20.0 nmol mL^−1^ for GSH and Hcy; 0.5, 2.0, and 4.0 nmol mL^−1^ for α-LA; 5.0, 10.0, and 150.0 nmol mL^−1^ for NAC; and 5.0, 10.0, and 25.0 mg mL^−1^ for HSA. The rest of the procedure was the same as in Section 3.5.1, and all the analyses were performed in triplicate. The next step was to evaluate the matrix effect in plasma samples from five different subjects at the same analyte concentrations and using the same protocol as above. The slopes of the calibration curves from the standard addition experiments were then compared for all the analytes.

### 3.6. Application to Real Samples

The content of total thiols in plasma samples from 10 potentially healthy volunteers was estimated. The method of single standard addition was used. For this purpose, measurements of the analytical signal were made for the plasma samples containing only the analyte and for the plasma samples with the addition of a known amount of the standard at concentrations of 50.0 nmol mL^−1^ for Cys and NAC, 20.0 nmol mL^−1^ for Cys-Gly, GSH, and Hcy, 4.0 nmol mL^−1^ for α-LA, and 10.0 mg mL^−1^ for HSA. The procedure was the same as presented in Section 3.5.1.

### 3.7. Statistical Analysis

All calculations, graphs, and statistical analyses were performed using Microsoft Excel 16.0 (Microsoft Corporation). Each value in the calibration charts represents the mean of three independent measurements with the standard deviations indicated. Unless otherwise noted, the graphs show a representative set of results from a plasma sample obtained from a single individual. All the results were presented as the means ± SD of three chromatographic runs. Linear regression was applied to develop an equation for predicting thiol concentration in plasma. Linear least-squares regression was used to calculate the linearity relationship between peak ranges and analyte concentrations.

## 4. Conclusions

In recent years, the detection of biothiols has attracted considerable interest because of their central role in a variety of physiological and pathological processes in the human body [52]. Continuous monitoring of the concentrations of endogenous thiols in biological fluids is extremely important to determine their content changes during disease development. Therefore, it can be a valuable source of information when assessing a patient’s health status. HPLC-FLD is the most widely used method for the determination of endogenous thiols in biological samples due to its high sensitivity and robustness [14]. In this work, we propose a new, sensitive, and simple protocol for the simultaneous determination of HSA and six low-molecular-weight thiols in human plasma. The assay is based on the reduction of disulfide bonds with NaBH_4_ followed by pre-column derivatization with mBBr. The thiol–bimane derivatives are then separated and quantified by RP-HPLC with fluorescence detection at the excitation and emission wavelengths of 378 nm and 492 nm, respectively. The major advantage of the presented method is that it allows the simultaneous determination of compounds with very different physicochemical properties in a complex matrix at the same analytical wavelengths and in a relatively short time of 35 min. The method was validated according to the FDA [65] and ICH [66] guidelines and the recovery, accuracy, and precision values meet the criteria for the analysis of biological samples. The devised method can be successfully used to determine HSA, α-LA, GSH, Cys, Hcy, Cys-Gly, and NAC concentrations in plasma samples derived from potentially healthy individuals.

## Figures and Tables

**Table 1:** Validation data.

Analyte	Regression Equation	R^2^	Linear Range (nmol mL^−1^)	Precision (%)		Recovery (%)		LOD (nmol mL^−1^)	LOQ (nmol mL^−1^)
				Min.	Max.	Min.	Max.		
HSA	y = 150,925x + 10,000,000	0.9953	1.76–30.0 ^a^	0.40	5.80	92.94	113.67	0.59 ^a^	1.76 ^a^
α-LA	y = 3487.6x + 4187.2	0.9977	0.29–5.0	0.26	6.48	83.38	106.88	0.10	0.29
GSH	y = 6153x + 18,335	0.9991	1.16–35.0	0.24	10.92	84.71	99.15	0.39	1.16
Cys	y = 6765.9x + 806,715	0.9994	9.83–450.0	0.89	6.24	87.27	110.74	3.28	9.83
Hcy	y = 6621.9x + 34,488	0.9994	0.55–40.0	0.90	11.93	83.89	115.77	0.18	0.55
NAC	y = 34,376x + 379.58	0.9999	0.34–50.0	0.17	1.26	81.87	106.07	0.11	0.34
Cys-Gly	y = 3370.3x + 54,775	0.9998	1.45–45.0	0.90	5.33	96.34	114.56	0.48	1.45

^a^ The unit of measurement for linear range, LOD and LOQ for HSA is mg mL^−1^.

**Table 2:** Intra-day and inter-day precision and accuracy evaluation for thiols in human plasma samples based on the proposed method, *n* = 3.

Analyte	Concentration (nmol mL^−1^)	Precision (%)		Accuracy (%)	
		Intra-Day	Inter-Day	Intra-Day	Inter-Day
HSA ^a^	1.76	0.35	1.49	119.48	99.31
	5.0	0.10	4.83	96.77	109.77
	15.0	0.35	1.99	97.49	94.78
	30.0	0.37	2.47	100.65	101.05
α-LA	0.29	0.55	0.18	97.16	84.30
	0.9	0.34	0.03	104.90	95.83
	1.75	0.60	7.34	97.31	107.24
	4.0	0.20	9.18	100.28	98.91
GSH	1.16	1.50	2.87	89.10	116.57
	4.0	0.71	1.25	100.67	100.87
	15.0	0.05	0.28	101.31	97.14
	30.0	0.13	0.22	99.68	100.68
Cys	9.83	2.23	4.25	85.56	97.18
	30.0	2.66	2.22	93.51	115.62
	100.0	0.57	6.94	104.43	94.38
	350.0	0.10	10.71	99.70	100.35
Hcy	0.55	3.85	14.57	97.45	82.17
	1.7	5.34	2.55	88.30	90.84
	15.0	0.43	0.27	102.69	103.24
	30.0	0.01	3.69	99.37	99.22
NAC	0.34	0.25	0.11	85.24	98.98
	1.0	0.48	2.05	100.83	115.95
	20.0	0.29	0.73	100.42	98.48
	40.0	0.04	0.10	99.90	100.37
Cys-Gly	1.45	1.43	5.09	108.52	93.58
	4.5	0.19	1.58	85.16	82.99
	20.0	0.19	0.36	105.41	108.83
	35.0	0.70	0.19	98.46	97.41

^a^ Unit of measurements for HSA is mg mL^−1^.

**Table 3:** Total low-molecular-weight thiols (nmol mL^−1^) and HSA (mg mL^−1^) in plasma from 10 subjects, *n* = 3.

No.	HSA		GSH		Cys		Hcy		Cys-Gly	
	Mean (SD)	RSD (%)	Mean (SD)	RSD (%)	Mean (SD)	RSD (%)	Mean (SD)	RSD (%)	Mean (SD)	RSD (%)
1	42.62 (3.01)	7.06	4.14 (0.25)	6.00	65.54 (2.08)	3.22	3.07 (0.28)	9.05	30.71 (0.29)	0.96
2	17.58 (1.51)	8.61	7.93 (0.67)	8.44	54.11 (3.06)	5.66	2.99 (0.11)	3.70	62.55 (3.40)	5.43
3	11.73 (0.72)	6.11	5.80 (0.28)	4.85	74.72 (7.48)	10.01	4.76 (0.22)	4.59	46.49 (4.98)	10.71
4	40.34 (2.64)	6.53	6.00 (0.40)	6.67	89.34 (4.60)	5.14	5.46 (0.54)	9.95	80.28 (3.14)	3.91
5	10.71 (0.49)	4.56	4.77 (0.22)	4.64	67.38 (5.56)	8.25	3.72 (0.25)	6.67	55.48 (4.38)	7.89
6	13.63 (1.25)	9.18	5.22 (0.33)	6.29	70.00 (3.38)	4.83	3.42 (0.14)	4.03	90.16 (7.00)	7.76
7	14.92 (0.84)	5.64	5.25 (0.23)	4.32	36.85 (3.55)	9.64	2.48 (0.07)	2.62	42.90 (1.40)	3.27
8	24.99 (1.02)	4.08	5.69 (0.24)	4.21	53.95 (4.28)	7.94	5.47 (0.48)	8.83	45.75 (3.14)	6.86
9	38.12 (3.18)	8.33	5.33 (0.41)	7.66	41.59 (3.31)	7.95	3.32 (0.31)	9.23	19.63 (1.83)	9.31
10	25.15 (1.31)	5.22	2.73 (0.10)	3.79	77.44 (5.79)	7.48	4.56 (0.45)	9.83	28.08 (3.22)	11.48

**Figure A1:**
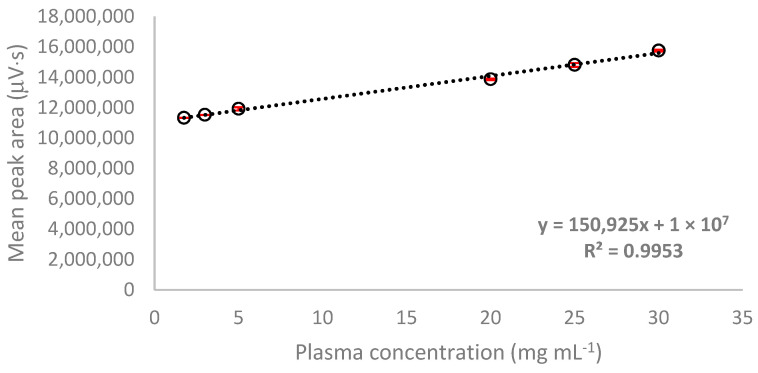
Calibration curve for human serum albumin.

**Figure A2:**
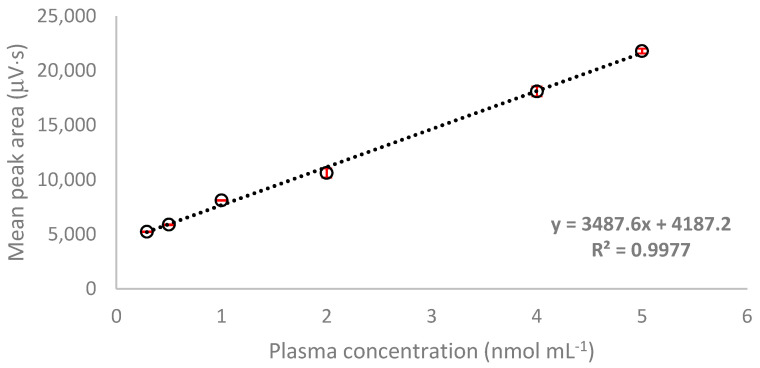
Calibration curve for α-lipoic acid.

**Figure A3:**
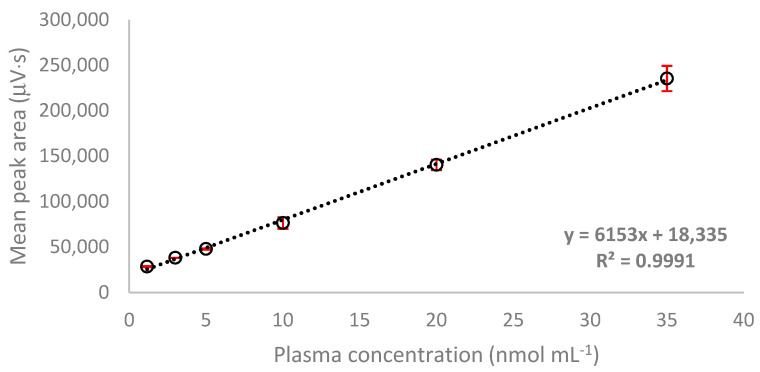
Calibration curve for glutathione.

**Figure A4:**
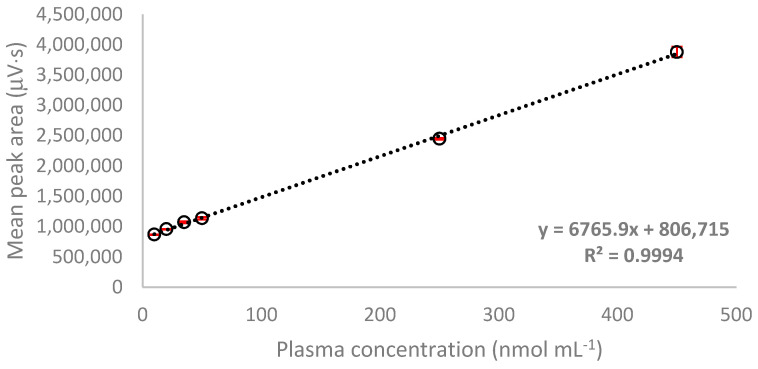
Calibration curve for cysteine.

**Figure A5:**
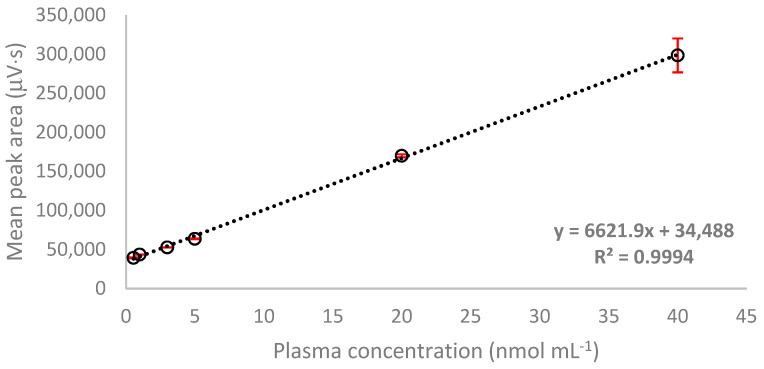
Calibration curve for homocysteine.

**Figure A6:**
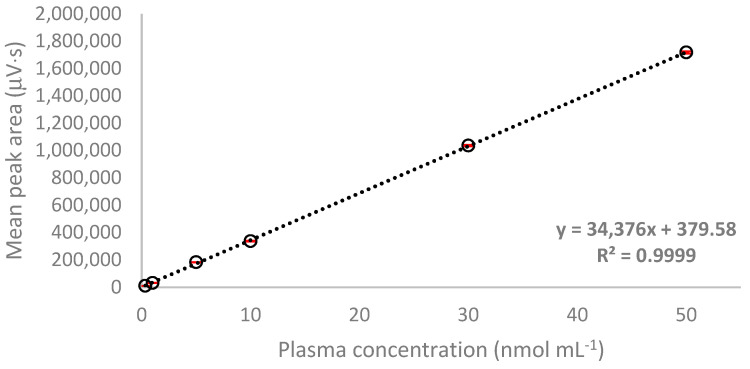
Calibration curve for *N*-acetyl-l-cysteine.

**Figure A7:**
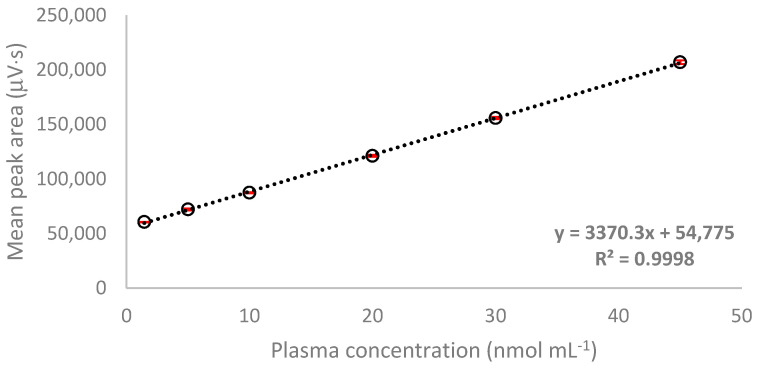
Calibration curve for cysteinylglycine.

## Data Availability

HPLC data are available from the authors.

## Supplementary Materials

The following are available online, Figure S1: Dependence of the mean peak area on the pH of the buffer used for the derivatization reaction, *n* = 3, Figure S2: Dependence of the mean peak area on the volume of mBBr, *n* = 3, Figure S3: Dependence of the mean peak area on the time of the derivatization reaction, *n* = 3.

## Sample Availability

Plasma samples were damaged during the experiments and are not available from the authors.
