# Supplementary material for: Simultaneous Determination of Human Serum Albumin and Low-Molecular-Weight Thiols after Derivatization with Monobromobimane
Source: Molecules. 2021 Jun 1;26(11):3321. doi: 10.3390/molecules26113321 (PMC8198679; doi:10.3390/molecules26113321)
Supplement: Supplementary file 1 [file molecules-26-03321-s001.zip › molecules-1214859-supplementary.pdf]

Simultaneous determination of human serum albumin and low-molecular-weight thiols after derivatization with monobromobimane

Katarzyna Kurpet [1,2,*], Rafał Głowacki [2] and Grażyna Chwatko [2,*]

[1] University of Lodz, Doctoral School of Exact and Natural Sciences, Banacha 12/16, 90-237 Lodz, Poland; katarzyna.kurpet@edu.uni.lodz.pl
[2] University of Lodz, Faculty of Chemistry, Department of Environmental Chemistry, Pomorska 163, 90-236 Lodz, Poland; rafal.glowacki@chemia.uni.lodz.pl (R.G.); grazyna.chwatko@chemia.uni.lodz.pl (G.C.)
* Correspondence: grazyna.chwatko@chemia.uni.lodz.pl; phone: +48 42 635 58 43 (G.C.); katarzyna.kurpet@edu.uni.lodz.pl; phone: +48 42 635 58 03 (K.K.)

Supplementary materials

The yield of the derivatization reaction was optimized with respect to pH, excess reagent, and time. The influence of buffer pH on the analytical signal (Figure S1) was tested using the following procedure: to a polypropylene tube 60 µl of analyte mixture was added to obtain the following final concentrations: 10 nmol mL^{-1} Cys, Hcy, NAC, Cys-Gly, GSH, α-LA, and 3 mg mL^{-1} HSA. Then, 20 µl of n-octanol, 30 µl of reducer, and 6 µl of 3 mol L^{-1} HCl were added to the test tube. After 5 min, 677 µl of 0.2 mol L^{-1} buffer TRIS-HCl containing 0.2 mmol L^{-1} EDTA at following pH 7.5, 8.0, 9.0, 9.5, 10.0, 10.5 was added. Subsequently, a derivatization reaction was performed with 7 µl of 0.1 mol L^{-1} mBBr. After 10 min, 200 µl of 1 mol L^{-1} HCl was added to the tube. Of the final sample, 5 µl was injected into the chromatographic column.

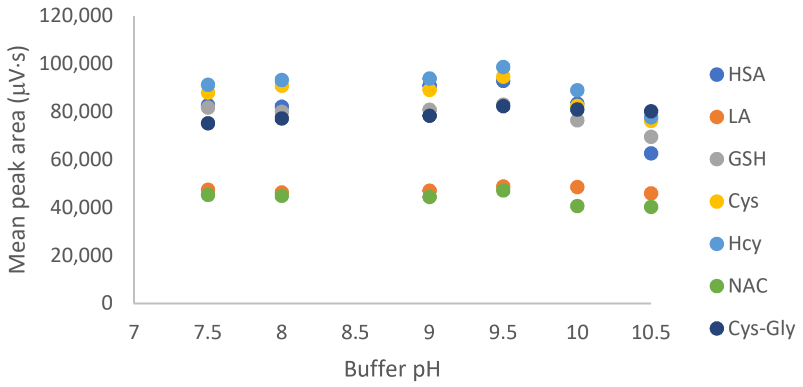

Figure S1. Dependence of the mean peak area on the pH of the buffer used for the derivatization reaction, $n = 3$.

The influence of mBBr volume on the analytical signal (Figure S2) was tested using the procedure described above. The derivatization reaction was carried out in appropriate amount of 0.2 mol L^{-1} TRIS-HCl with 0.2 mmol L^{-1} EDTA buffer pH 9.5 for 20 min using the following volumes of 0.1 mol L^{-1} mBBr: 1, 2, 5, 7, 10, 15 µL.

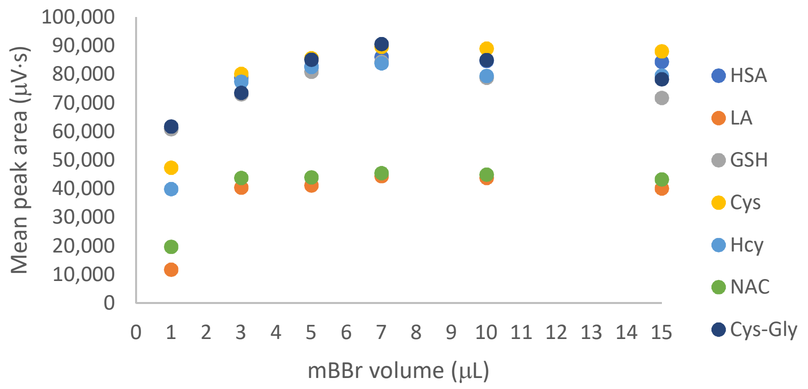

Figure S2. Dependence of the mean peak area on the volume of mBBr, $n = 3$.

The last parameter tested was the kinetics of derivatization (Figure S3). The procedure was the same as described above. The derivatization reaction was carried out in 0.2 mol L^{-1} TRIS-HCl with 0.2 mmol L^{-1} EDTA buffer pH 9.5 for: 5, 10, 15, 20, 30, 45 and 60 minutes.

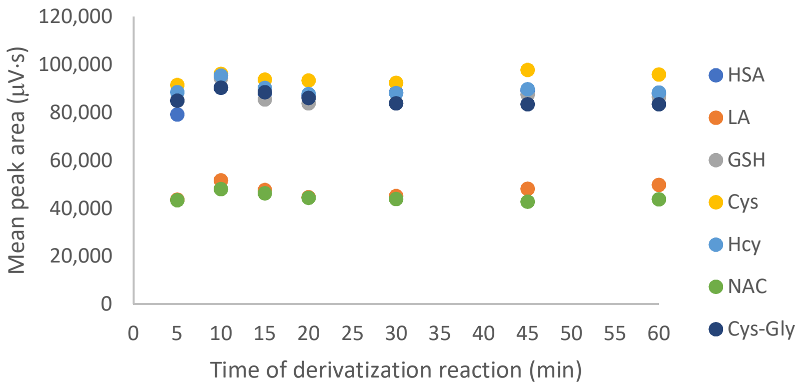

Figure S3. Dependence of the mean peak area on the time of derivatization reaction, $n = 3$.
